# The Spatial Predilection for Early Esophageal Squamous Cell Neoplasia: A “Hot Zone” for Endoscopic Screening and Surveillance

**DOI:** 10.1097/MD.0000000000003311

**Published:** 2016-04-18

**Authors:** Wen-Lun Wang, I.-Wei Chang, Chien-Chuan Chen, Chi-Yang Chang, Jaw-Town Lin, Lein-Ray Mo, Hsiu-Po Wang, Ching-Tai Lee

**Affiliations:** From the Department of Internal Medicine (W-LW, C-YC, J-TL, L-RM, C-TL) and Department of Pathology, E-Da Hospital/I-Shou University, Kaohsiung (I-WC); Department of Internal Medicine, National Taiwan University Hospital, Taipei (C-CC, J-TL, H-PW); and School of Medicine, Fu Jen Catholic University, New Taipei (J-TL), Taiwan.

## Abstract

Early esophageal squamous cell neoplasias (ESCNs) are easily missed with conventional white-light endoscopy. This study aimed to assess whether early ESCNs have a spatial predilection and the patterns of recurrence after endoscopic treatment.

We analyzed the circumferential and longitudinal location of early ESCNs, as well as their correlations with exposure to carcinogens in a cohort of 162 subjects with 248 early ESCNs; 219 of which were identified by screening and 29 by surveillance endoscopy. The circumferential location was identified using a clock-face orientation, and the longitudinal location was identified according to the distance from the incisor.

The most common circumferential and longitudinal distributions of the early ESCNs were found in the 6 to 9 o’clock quadrant (38.5%) and at 26 to 30 cm from the incisor (41.3%), respectively. A total of 163 lesions (75%) were located in the lower hemisphere arc, and 149 (68.4%) were located at 26 to 35 cm from the incisor. One hundred eleven (51%) early ESCNs were centered within the “hot zone” (i.e., lower hemisphere arc of the esophagus at 26 to 35 cm from the incisor), which comprised 20% of the esophageal area. Exposure to alcohol, betel nut, or cigarette was risk factors for the development of early ESCNs in the lower hemisphere. After complete endoscopic treatment, the mean annual incidence of metachronous tumors was 10%. In addition, 43% of the metachronous recurrent neoplasias developed within the “hot zone.” Cox regression analysis revealed that the index tumor within the hot zone (hazard ratio [HR]: 3.19; 95% confidence interval [CI]: 1.17–8.68; *P* = 0.02) and the presence of numerous Lugol-voiding lesions in the esophageal background mucosa were independent predictors for metachronous recurrence (HR: 4.61; 95% CI: 1.36–15.56; *P* = 0.01).

We identified a hot zone that may be used to enhance the detection of early ESCNs during endoscopic screening and surveillance, especially in areas that lack resources and have a high prevalence of ESCNs.

## INTRODUCTION

Esophageal cancer is a common and highly lethal malignancy, causing more than 400,000 deaths/y worldwide.^1^ In the Asia-Pacific region, esophageal squamous cell neoplasia (ESCN) is the major form of the disease, and the incidence continues to rise.^1,2^ Despite recent advances in the diagnosis and management of this lethal cancer, the 5-year survival rate remains <30%.^1–3^ Importantly, patients with esophageal cancer are almost always diagnosed at a late stage,^3,4^ due to the lack of effective screening programs and easily overlooked.^5–7^ Early ESCNs, including high-grade dysplasia (HGD) and intra-mucosal cancer, usually appear as flat lesions with minimal color change, and thus are difficult to be detected with conventional white-light endoscopy.^8,9^ Moreover, ESCNs are more prevalent in developing countries where the socioeconomic status is relatively poor, and thus expensive image-enhanced devices^10,11^ are not commonly used leading to the ESCNs almost always being diagnosed at a late stage. Therefore, the identification of a highly susceptible area where early ESCNs are most likely to develop and recur will enhance their detection and be clinically beneficial for endoscopic screening and surveillance.

Patients who drink alcohol, chew betel nut, smoke cigarettes and have a prior history of head and neck squamous cell carcinoma have been reported to be predisposed to developing ESCNs.^12,13^ Anatomic factors may play a role in the location of lesions due to differing levels of exposure to carcinogens. However, there is currently limited information with regard to the carcinogenetic impact on anatomical subsites of the esophagus. Whether early ESCNs have a predilection for a particular spatial location and the pattern of metachronous recurrence after endoscopic treatment are still unknown. The aim of this study was to determine whether early ESCNs have a predilection for a particular area within the esophagus, and whether this is associated with exposure to carcinogens. In addition, we also investigated the pattern of recurrence after complete endoscopic therapy.

## MATERIALS AND METHODS

### Patients and Design

Since 2008, we have used narrow-band imaging and Lugol chromoendoscopy to screen individuals at high risk of esophageal cancer, including those with a history of head and neck cancer^11,13,14^ and those with well-established risk factors including drinking alcohol, chewing betel nut, and smoking cigarettes. Based on this cohort and the patients who were incidentally detected during routine endoscopic examinations, we consecutively recruited adults with newly diagnosed histologically proven early stage ESCNs (squamous HGD, intramucosal cancer [ImCa]) at E-Da Hospital, Taiwan, from June 2008 to May 2014. All of the enrolled subjects received magnifying endoscopy and endoscopic ultrasound to evaluate the tumor invasion depth and confirm the early stage of tumor.^15,16^ Patients having a stricture that prevented passage of an endoscope or a history of endoscopic resection, surgery, or radiation of the esophagus were excluded. We then retrospectively review the endoscopic photographs of the circumferential and longitudinal location of early stage ESCNs from this consecutive cohort. The study protocol conformed to the 1975 Declaration of Helsinki and was approved by the institutional review boards of EDa Hospital (EMRP-46103N). Each patient signed their written informed consent to participate in this study.

### Data Collection

Demographic characteristics, social habits, substance use, and medical history were collected via an interview with the participants using a standardized questionnaire. Alcohol drinkers, betel quid chewers, and cigarette smokers were defined as those consuming any alcoholic beverage during the week, those who chewed >7 betel quids/wk, and those who smoked >10 cigarettes/wk for at least 6 months, respectively.^13^ The patients with a history of head and neck tumors were grouped by location: oral cavity, oropharynx, hypopharynx, and larynx. After meticulously screening the esophagus with image-enhanced endoscopy including narrow-band imaging and Lugol chromoendoscopy, an endoscopic biopsy and endoscopic ultrasound were done for all suspected lesions. The number and multiform pattern of Lugol-voiding lesions in the esophageal background mucosa were also recorded.^17,18^ Both static photographs and video clips were taken during the procedure.

**FIGURE 1:**
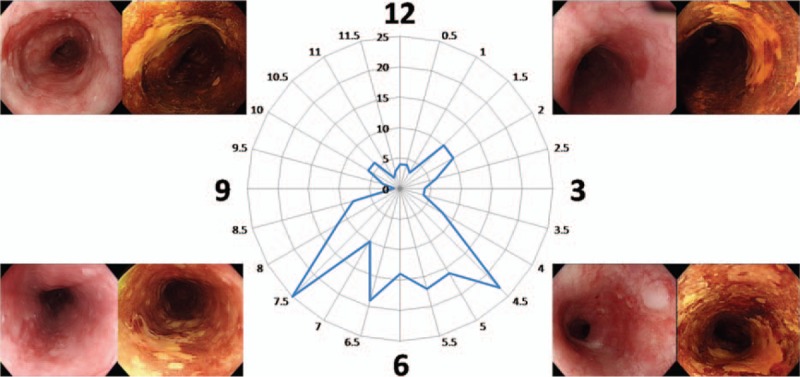
Circumferential distribution (clock face) of early stage esophageal squamous cell neoplasia.

The circumferential location of the early ESCNs was identified using a clock-face orientation (Figure 1) and further divided into 4 quadrants. If the lesion occupied the whole circumference of the esophagus, it will be excluded from circumferential distribution analysis. The longitudinal location was identified according to the distance from the incisor, and then classified into 5 sections: inlet to 20 cm, 21 to 25 cm, 26 to 30 cm, 31 to 35 cm, and 36 cm to esophagocardiac junction. In those cases where a lesion encompassed more than 2 portions, the central point of the lesion was used to determine the predominant location involved.

All spatial locations found within the endoscopic photographs were recorded by a single endoscopist (W-LW), with the endoscope in a neutral position and the patient in the left lateral decubitus position. Thus, the lower hemisphere arc of the endoscopic view (the 2nd and 3rd quadrants) consisted of the posterior esophageal wall. If the lesion spanned 2 or more quadrants, the central portion of the lesion (i.e., the midpoint) was used to designate its position.

### Endoscopic Surveillance and Outcome Measures

After endoscopic treatment with endoscopic submucosal dissection or radiofrequency ablation, the patients received surveillance endoscopy with image-enhancement including Lugol staining and narrow-band imaging every 6 months. The primary endpoint was tumor recurrence after complete treatment. Metachronous tumor recurrence was defined as a tumor recurring at a new site after more than 6 months of complete remission status.

### Statistical Analysis

All statistical analyses were performed using SPSS software (version 18.0; SPSS Inc., Chicago, IL). Patient data were presented as means, standard deviations, and percentages. A chi-squared test was used to test the distribution of lesions in the upper and lower hemispheres from uniformity in the distribution of lesions. We performed subgroup analysis by separating statistics of HGD and ImCa to compare the differences between precancerous and cancerous lesions. The cumulative recurrence-free survival rates were estimated using Kaplan–Meier curves and assessed using 2-tailed log-rank tests. Univariate and multivariate Cox proportional hazard analyses were performed to determine the independent risk factors for metachronous recurrence. A *P* value <0.05 was considered to indicate statistical significance.

## RESULTS

### Patients and Endoscopic Characteristics

**FIGURE 2:**
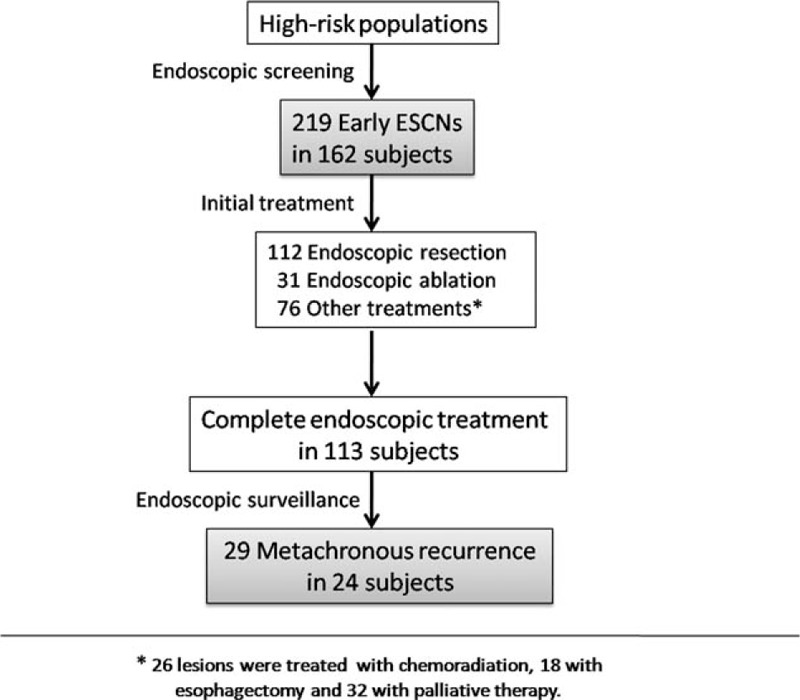
Flow chart of patient enrollment for analysis.

A total of 162 subjects with 248 early ESCNs, of which 219 lesions were detected by screening and 29 by surveillance endoscopy, were enrolled in this study (Figure 2). The clinical and endoscopic characteristics are shown in Table 1. One hundred fifty-four patients were male with average age of 52.65 years (range: 30–87 years) at the time of detection. One hundred eleven patients (68.5%) had a history of head and neck cancers, and 44 patients (27.1%) had multiple (≥2) lesions in the esophagus. Among the 248 lesions, 157 occupied less than half of the circumference of the esophagus, and 30 (12.1%) occupied the whole circumference. The average neoplastic size was 33.2 mm (range: 5–170 mm).

**TABLE 1:**
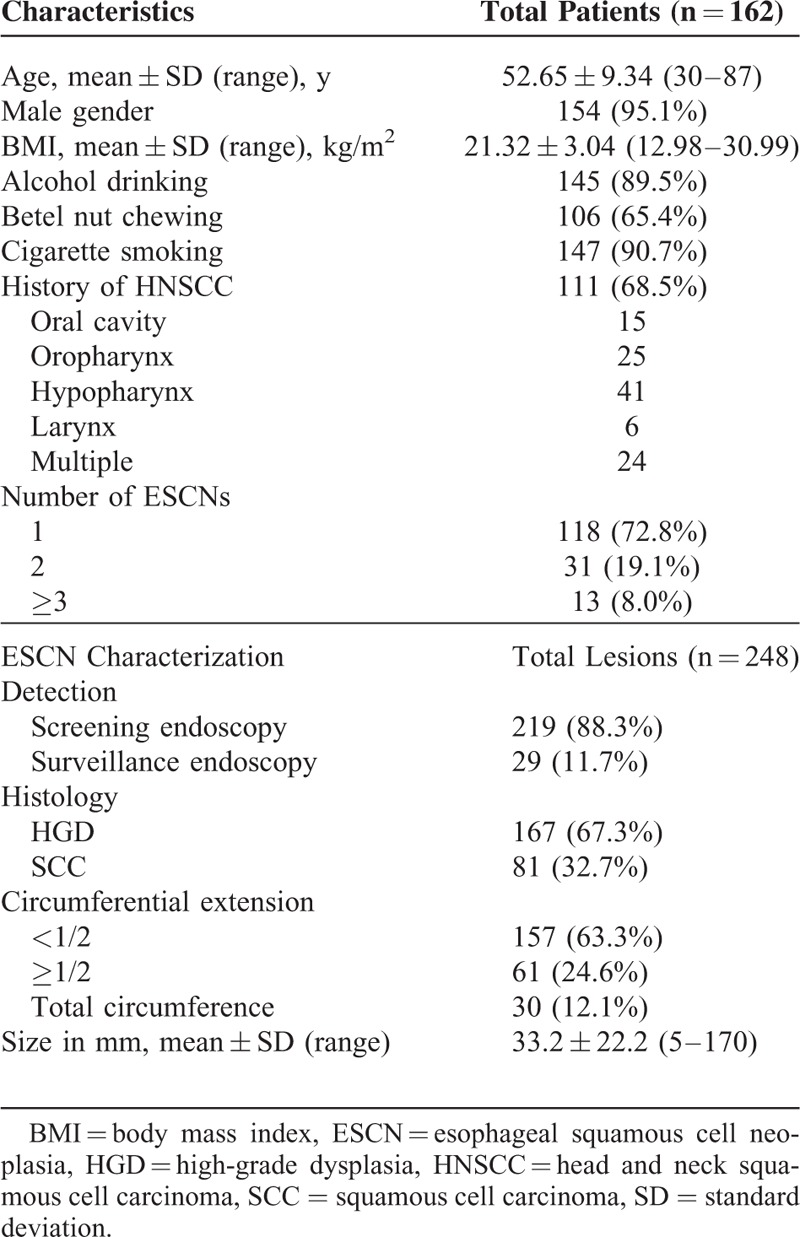
Patients Demographics and Tumor Characteristics

### Spatial Distribution of Early Esophageal Squamous Cell Neoplasia

**FIGURE 3:**
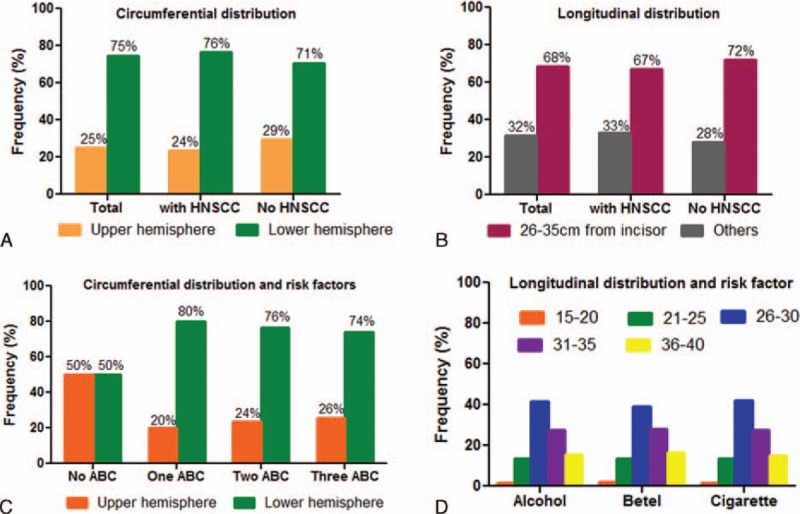
Relationship between spatial distribution and risk factor exposure. (A) The circumferential distribution and (B) longitudinal distribution of early ESCNs in patients with or without a history of head and neck cancer. (C) The association between circumferential distribution and alcohol, betel nut, cigarette exposure. (D) The association between longitudinal distribution and alcohol, betel nut, cigarette exposure. ABC = alcohol, betel nut, cigarette, ESCN = esophageal squamous cell neoplasias, HNSCC = head and neck squamous cell carcinoma.

The circumferential distribution (clock face) of the location of the early ESCNs is shown in Figure 1 and Table 2. Overall, the highest percentage of early ESCNs was found in the 6 to 9 o’clock quadrant (38.5%), followed by the 3 to 6 o’clock quadrant (36.2%). Similar findings were found for both high-grade squamous dysplasia and ImCa or screening and surveillance endoscopy. There was a significantly higher rate of early ESCNs (HGD or ImCa) in the lower hemisphere of the endoscopic view (2nd and 3rd quadrants) compared with the upper hemisphere (74.7% vs 25.3%, *P* < 0.001; Figure 3A).

**TABLE 2:**
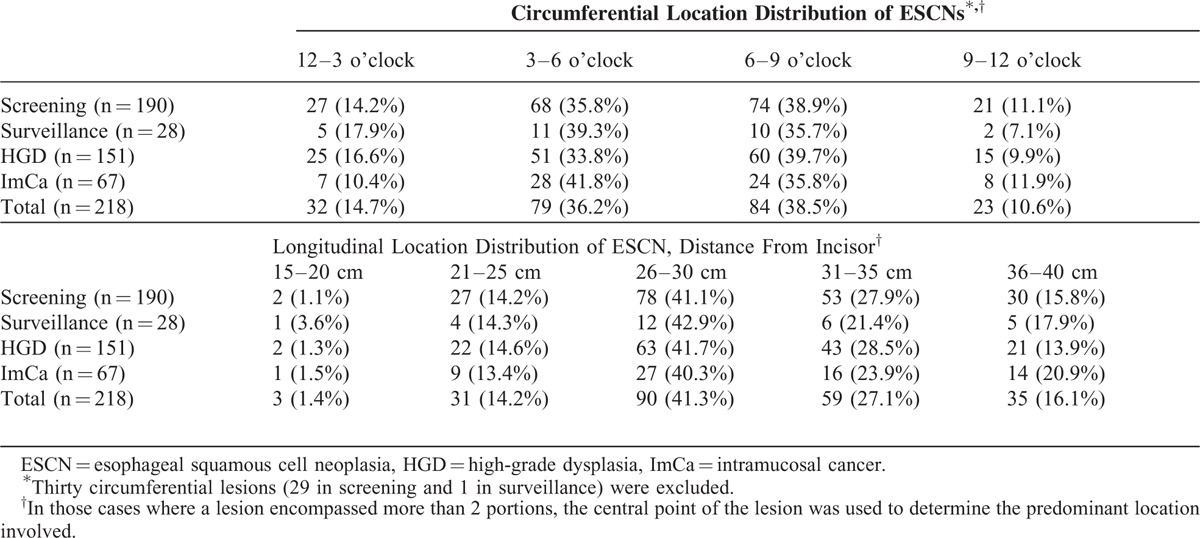
Spatial Distribution of Early ESCNs

The longitudinal distribution is shown in Table 2 and Figure 3B. Overall, the highest percentage of early ESCNs was found at 26 to 30 cm from the incisor (41.3%), followed by 31 to 35 cm (27.1%). There was a significantly higher rate of early ESCNs (HGD or ImCa) located at 26 to 35 cm compared with other segments (68.4% vs 31.6%, *P* < 0.001). Similar findings were found in both screening and surveillance.

Accordingly, 111 (51%) early ESCNs were centered within the “hot zone,” which was the lower hemisphere arc of the esophagus at 26 to 35 cm from the incisor, comprising 20% of the esophageal area.

### Associations Between Risk Factors and Spatial Distribution of Early ESCNs

The relationships between risk factors and the circumferential distribution of ESCNs are shown in Figure 3. Patients, with history of head and neck cancers or not, did not show significant association with spatial distribution of ESCNs (Figure 3A and B), with 76% and 71% at the lower hemisphere; 67% and 72% at 26 to 35 cm from the incisor, respectively. In the patients who did not drink alcohol, chew betel nut, or smoke cigarettes, the distributions were equal over both the upper and lower hemisphere. However, when the patients were exposed to more than 1 of these well-established risk factors, the distributions tended to develop in the lower hemisphere (>74%; Figure 3C). With regard to tumor longitudinal distribution, the patients who were exposed to anyone of the risk factors tended to develop ESCNs over the 26 to 30 and 31 to 35 cm region of the esophagus from the incisor (Figure 3D).

### Pattern and Predictors of Metachronous Neoplastic Recurrence

**FIGURE 4:**
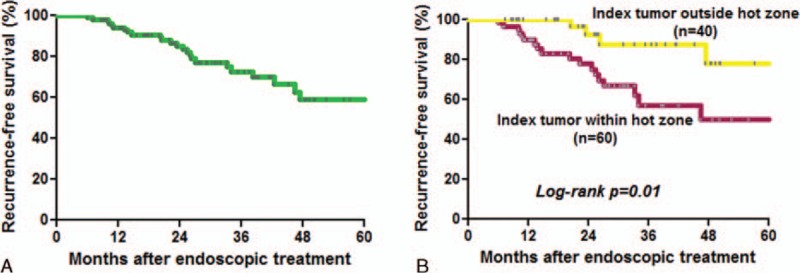
(A) Recurrence-free survival curve of patients who received complete endoscopic therapy. (B) The patients with the index tumor within the hot zone had a poorer recurrence-free survival than those without (*P* = 0.01).

Among the 219 early ESCNs, 143 received endoscopic therapy (112 resections and 31 ablations) as the initial treatment, and 76 received other treatment modalities (18 surgery, 26 chemoradiation, and 32 palliative). Among those who received endoscopic therapy, 113 patients achieved complete remission after treatment (R0 resection or total ablation). During the follow-up period (mean period: 26.6 months; range: 7–78 months) with surveillance endoscopy, 29 lesions in 24 subjects (21.2%) developed metachronous recurrence. All of the metachronous tumors were early stage ESCNs. The recurrence-free survival curve is shown in Figure 4A. The cumulative metachronous recurrence rate at 3 years was 30%, and the mean annual incidence of newly diagnosed tumors was 10%. One patient had tumor recurrence that occupied the whole circumference. The pattern of recurrent tumor distribution is demonstrated in Table 2. Similarly, 75% of neoplastic recurrences were located at the lower hemisphere, and 64.3% were located at 26 to 35 cm. Of 28, 12 (43%) metachronous recurrent neoplasias developed within the “hot zone.” The patients with the index tumor in the hot zone had a poorer recurrence-free survival (*P* = 0.01; Figure 4B). The cumulative metachronous recurrence rates at 3 years in the patients with and without the index tumor in the hot zone were 45.0% and 12.5%, respectively. Cox regression hazard analysis revealed that the index tumor located within the hot zone (hazard ratio [HR]: 3.19; 95% confidence interval [CI]: 1.17–8.68; *P* = 0.02) and the presence of numerous Lugol-voiding lesions in the esophageal background mucosa were independent predictors for metachronous neoplastic recurrence (HR: 4.61; 95% CI: 1.36–15.56; *P* = 0.01, Table 3).

**TABLE 3:**
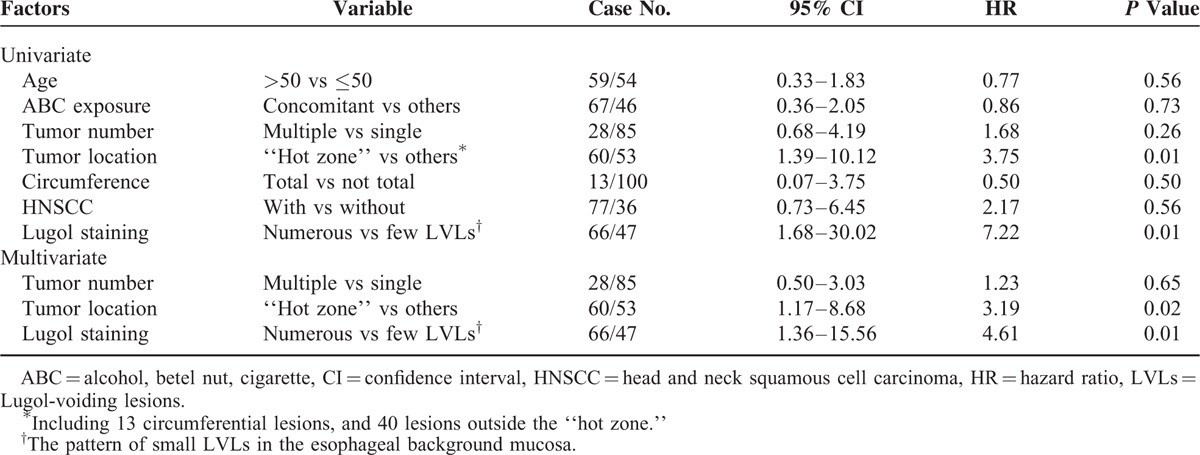
Cox Hazard Regression to Predict Metachronous Tumor Recurrence

## DISCUSSION

The incidence of esophageal cancer is increasing worldwide, especially in Eastern countries. Most patients are diagnosed at a late stage which may be because early ESCNs are easily missed with conventional endoscopy, and also because appropriate screening and surveillance strategies are lacking. Esophageal squamous cell carcinomas are more prevalent in developing countries and poor socioeconomic regions.^1^ The relatively poor socioeconomic status in these regions means that expensive image-enhanced modalities are not widely available, so that the ESCNs are almost always diagnosed at a late stage with a poor prognosis. Our study demonstrated that early ESCNs had a spatial and longitudinal predilection, and we identified a hot zone that may be used to enhance the detection of early ESCNs during endoscopic screening and surveillance. We also identified the risk factors and pattern of metachronous recurrence over the esophagus after complete endoscopic treatment.

Our findings are novel and we believe that this would have a clinical implication for endoscopic screening and surveillance of esophageal cancer in high-risk patients. We suggest that endoscopists should pay more attention to this hot area with conventional white-light endoscopy due to more than 50% of the early ESCNs developed within this small area (comprising only 20% of the esophageal surface).

To the best of our knowledge, this study is the first to analyze the circumferential spatial predilection of early ESCNs. The possible mechanisms for the hot zone may be due to anatomic factors or exposure to carcinogens. We found that the patients who were exposed to at least 1 kind of carcinogen were predisposed to develop ESCNs over the lower hemisphere arc in endoscopic view; that is, the posterior esophageal wall or dependent part while in the supine position. We speculate that carcinogens will gradually accumulate at the dependent part of the esophagus after repeated exposure, resulting in esophageal carcinogenesis. In addition, we found that early ESCNs frequently developed over the region 26 to 35 cm below the incisor. Previous studies have reported similar findings in that ESCNs frequently develop in the middle-third of the esophagus; however, these studies generally enrolled patients with advanced stage cancers.^19–21^ We found that the patients who drank alcohol, smoked cigarettes, or chewed betel nut were all predisposed to developing cancer within the area 26 to 35 cm below the incisor (Figure 3D). However, the mechanism is still uncertain. A different origin of the blood supply to the esophagus has been reported to be a possible reason.^22^ The upper- and lower-thirds of the esophagus are supplied by the inferior thyroid artery and branches of the left gastric artery, respectively, and they are smaller than the branches from the descending thoracic aorta which supplies the middle-third of the esophagus. The higher risk of developing cancer in the middle-third of the esophagus may be related to more abundant blood supply in this area, thus increasing the action of tobacco and alcohol carcinogens in this region. Moreover, recent studies have reported that this region, composed of transition from striated to smooth muscle, was associated with diminished peristalsis.^23–25^ The stasis of swallowed boluses across the transition zone may partly explain why the carcinogens accumulate within the middle-third of the esophagus.

In our cohort, the patients with early ESCNs who received complete endoscopic therapy had a high prevalence of metachronous tumor recurrence (21.2%), suggesting a field cancerization effect in the esophagus.^26^ The cumulative metachronous recurrence rate at 3 years was 30%, and the mean annual incidence of newly diagnosed tumors was 10%, which are higher than in previous reports.^27,28^ This may be because we enrolled patients with a prior history of head and neck cancers and those with numerous small Lugol-voiding lesions in esophageal background mucosa, that may potentially have led to a higher incidence of esophageal cancer.^17,18^

In the present study, we detected 30 lesions occupying the whole esophageal circumference, and the centers of 23 (76.7%) of the tumors were located predominantly at 26 to 35 cm from the incisor. Such circumferential tumors have been reported to have a clinical impact on endoscopic resection, not only because they are difficult to approach therapeutically but also because they have a high stricture rate after complete resection.^11,29,30^ We thus tried to analyze the risk factors for the development of circumferential lesions by logistic regression analysis, and found that alcohol drinking tended to be associated with circumferential neoplasias with borderline statistical significance (odds ratio: 4.81; *P* = 0.15). However, the sample size is relatively small in this subgroup analysis, a large-scale study with dose–response relationship analysis is required to make a conclusion in the future.

There are several limitations to this study. First, the study is inevitably limited by its retrospective nature and further prospective studies are required to validate our results. Second, the endoscopic photographs were assessed by a single operator, which may result in misclassification biases if lesions were viewed by a misaligned endoscope. However, we believe that this influence was generally limited due to the consistency of trainings and practices of the endoscopists involved in this study. Also, the findings of saliva or Lugol solution pooling over the left side of image, provide an indirect evidence that the endoscope in a neutral position and the patient in the left lateral decubitus position. Therefore, every endoscopic report contained a set of images taken in the neutral position, thereby limiting the variations of the images. Third, the sample size of female subjects and substances nonuser are relatively too small to have a subgroup analysis of the spatial predilection in these populations. Fourth, the underlying mechanisms for the frequent development of early ESCNs within the hot zone are still not well-established, and further studies are needed to elucidate whether cancer stem cells play a role.

In conclusion, we identified a hot zone for the development of early ESCNs. We suggest that endoscopists should pay particular attention to the lower hemisphere and middle-third of the esophagus during endoscopic screening and surveillance, especially in areas that lack resources and have a high prevalence of ESCNs.
